# Quantification of Phytochemicals, Cellular Antioxidant Activities and Antiproliferative Activities of Raw and Roasted American Pistachios (*Pistacia vera* L.)

**DOI:** 10.3390/nu14153002

**Published:** 2022-07-22

**Authors:** Wang Yuan, Bisheng Zheng, Tong Li, Rui Hai Liu

**Affiliations:** 1 Overseas Expertise Introduction Center for Discipline Innovation of Food Nutrition and Human Health (111 Center), School of Food Sciences and Engineering, South China University of Technology, Guangzhou 510641, China; yuanwangyyy@163.com (W.Y.); febzheng@scut.edu.cn (B.Z.); 2 Guangdong ERA Food & Life Health Research Institute, Guangzhou 510670, China; 3 Department of Food Science, Cornell University, Ithaca, NY 14853, USA; tl24@cornell.edu

**Keywords:** pistachios, phytochemicals, antioxidant, antiproliferative activity

## Abstract

The consumption of pistachios has been linked to many potential health benefits. Phytochemicals in pistachios, including phenolics, vitamin E and carotenoids, have been considered to make contributions to the health benefits. The objectives of this study were (1) to explore the phytochemical profiles (total phenolics and total flavonoids, including both free and bound forms), selected phytochemicals, vitamin E and carotenoids of raw and roasted pistachios; (2) to determine total antioxidant activity and cellular antioxidant activity (CAA); and (3) to explore antiproliferative activities of pistachio extracts against human breast, liver and colon cancer cells in vitro. Both raw and roasted pistachios contained high total phenolics, at 479.9 ± 10.2 (raw) and 447.9 ± 9.4 (roasted) mg GAE/100 g, respectively, and high flavonoids, at 178.4 ± 10.6 (raw) and 144.1 ± 7.4 (roasted) mg GAE/100 g, respectively. The contributions of the free form to the total phenolics in pistachios were 82% (raw) and 84% (roasted), respectively, and the contributions of the free form to the total flavonoids in pistachios were 65% (raw) and 70% (roasted), respectively. Gentisic acid and catechin were the major phenolics in raw and roasted pistachios, respectively. Both raw and roasted pistachios had similar total antioxidant activity evaluated by Oxygen-Radical-Scavenging Capacity (ORAC) assay, at 7387.9 ± 467 (raw) and 7375.3 ± 602 (roasted) μmol TE/100 g, respectively. Both raw and roasted pistachio extracts exhibited cellular antioxidant activity inhibiting peroxyradical radical-induced oxidation, with CAA values of 77.39 ± 4.25 (wash) and 253.71 ± 19.18 (no wash) μmol QE/100 g of raw pistachios and 115.62 ± 3.02 (wash) and 216.76 ± 6.6 (no wash) μmol QE/100 g of roasted pistachios. Roasted pistachios contained more vitamin E when compared with raw pistachios, while raw pistachios contained more carotenoids than the roasted pistachios. Additionally, the free form of roasted pistachios extracts exhibited superior antiproliferation activity against HepG2, Caco-2 and MDA-MB-231 cancer cells in a dose-dependent manner, with EC_50_ 34.73 ± 1.64, 36.66 ± 3.3 and 7.41 ± 0.82 mg per mL, respectively. These results provided new knowledge about the phytochemical profiles, antioxidant activity, cellular antioxidant activity and antiproliferative activity of raw and roasted pistachios.

## 1. Introduction

The Mediterranean diet (MD), which consists of foods rich in antioxidants, phytochemicals and nutrients, has been considered as one of the most optimal diet patterns in the world. Many previous studies have demonstrated that MD is associated with risk reduction in CVD and cancers [1,2,3]. Nuts, as major components of MD, are rich in anticancer and antioxidant nutrients, including phytochemicals, vitamin E, fibers, phytosterols, and unsaturated fatty acids. Together with hazelnuts, walnuts, almonds and cashew nuts, pistachios are the one of the most consumed nuts by humans. Pistachios (*Pistacia vera* L.) are affiliated with the Anacardiaceae family and is the only commercial Anacardiaceae plant with the production of edible nuts [4]. Pistachios originated in western Asia, and dietary intake of pistachios is documented 300,000 years ago by the Neanderthals [5]. Currently, the United States is the leading producer of pistachios in the world, with 47% of world production, followed by Turkey (30%) and Iran (19%) [6]. California is the largest pistachio production location, with about 99% of the U.S. production. Ripe pistachios consist of three parts, kernels (seeds), skin (seed coat) and a hard shell outside. After the harvest, pistachios generally go through precleaning, dehulling, moisture reduction, roasting, and packaging. Roasting is a traditional process during pistachio production, which provides better aroma, taste, color and shine to the pistachios.

Pistachio consumption has been encouraged by health professionals as one of the components of healthy dietary patterns. These nuts are rich in healthy fats, including mono- and poly-unsaturated fatty acids and low saturated fatty acids. Furthermore, among widely consumed nuts, pistachios hold the supreme concentrations of potassium, γ-tocopherol, phytosterols, and xanthophyll carotenoids [7,8,9]. Additionally, pistachios have been listed among the 50 foods highest in antioxidant polyphenols [10]. Previous research [11] has shown a comprehensive phytochemical analysis of pistachios. They found fatty acids, phytosterols, and tocopherols were presented in lipophilic extracts from both the skin and kernel of pistachios. These lipophilic fractions significantly reduced lipid accumulation in mature adipocytes. Simona [12] et al. reported that there were significant amounts of xanthophyll carotenoids in pistachios (only the nut), namely lutein and zeaxanthin. Pistachios also contain substantial amounts of tocopherols and phenolic compounds. Phenolic compounds were the main water-soluble extracts, including anthocyanins, gallic acid, catechin, and quercetin. These are components with one or more aromatic rings and hydroxyl groups, including phenolic acids, flavonoids, stilbenes, coumarins and tannins [13].

Pistachios are not only excellent source of many important bioactive compounds, but also exhibit various health benefits to humans [14,15,16,17]. The various phenolics in pistachios are effective in stopping the formation of active oxygen species, providing protective effects against the oxidation of some indispensable biological macromolecules. At the same time, the consumption of pistachios has to do with reduced risk of mortality, total cancer, and all-cause mortality [18,19]. Some bioactive compounds, polyphenols and xanthophylls in pistachios have been suggested to present a rapid accessibility in simulated human digestion [12]. Recent studies show a significant negative association between the consumption of pistachios and cancer and other chronic disease risk factors [19,20]. Numerous studies have suggested that phytochemicals derived from pistachios potentially exhibit improved antioxidant activity and decreased the markers of oxidative stress in healthy subjects [21]. Additionally, these phytochemicals could take part in the antioxidant defense system through preventing the generation of some pro-oxidants, scavenging free radicals and reducing reactive oxygen intermediates [22].

Pistachios were suggested to have potential anticancer activities because of their antiproliferative activity [23]. It was reported that the fermentation supernatants of pistachios could inhibit LT97 colon adenoma cell proliferation in a dose-dependent manner [24], and the H_2_O_2_-induced DNA damage could be significantly reduced and levels of CAT mRNA increased by the fermentation supernatants of pistachios. The possible mechanism of action of the antiproliferative activity of pistachios has not been studied thoroughly. To date, most studies about the biological activities of pistachios have been centered on the lipid profile. A few studies have been concentrated on the phenolics of pistachio hulls and little attention has been focused on pistachios’ phytochemical profile, as well as the antioxidation and antiproliferation [18,25,26]. To fully understand the potential health benefits of pistachios, the objectives of this study were (1) to explore the phytochemical profiles (total phenolics and flavonoids, consisting of both free and bound forms), selected phytochemicals, vitamin E and carotenoids of raw and roasted pistachios; (2) to determine total antioxidant activity and cellular antioxidant activity (CAA); and (3) to determine antiproliferative activities of raw and roasted pistachio extracts against human breast, liver, and colon cancer cell lines in vitro.

## 2. Materials and Methods

### 2.1. Materials and Reagents

Folin–Ciocalteu reagents and other chemical regents such as fluorescein sodium salt, 2,2′-azobis (2-methylpropionamidine) dihydrochloride and standards for phytochemical profiles analysis were acquired from Sigma-Aldrich Chemical Co. (St Louis, MO, USA). Organic regents were purchased from ANPEL Scientific Instrument Co. (Shanghai, China). The standards for vitamin E and carotenoids profile analysis were provided by Wako Pure Chemical Industries (Tokyo, Japan) and Chromadex, Ltd. (Irvine, CA, USA).

Sample collection: The samples of American pistachios (raw and roasted), *Pistacia vera* L., were provided by American Pistachios Growers (Fresno, CA, USA). All samples were collected at least in triplicate in sealed plastic bottles or containers to avoid the moisture-loss-induced artifact, as described previously by our laboratory [27]. All collected samples were stored at −40 °C until use. The moisture contents of raw and roasted pistachios were 5.19 ± 0.05% and 3.23 ± 0.07%, determined by a normal hot-air oven method [28].

### 2.2. Cancer Cells and Cell Culture

Three cancer cell lines were selected in this study, including human liver cancer HepG2 cells, human colon cancer Caco-2 cells and human breast cancer MDA-MB-231 cells. All of them were all obtained from ATCC Co. (Manassas, VA, USA). These cells were cultivated in respective comfort medium such as Dulbecco’s Modified Eagle Medium (DMEM) (Gibco, Grand Island, NE, USA) or William’s E Medium (WME) (Gibco, Grand Island, NE, USA). Both media required the addition of nutrients such as fetal bovine serum and antibiotic/antimitotic. All cell cultures were incubated in an incubator with 5% CO_2_ at 37 °C.

### 2.3. Extraction of Soluble Free and Bound Phytochemical Compounds

The extractions of soluble free and bound phenolics of pistachios were achieved using the same method in our laboratory, with slight modification. Briefly, 4 g of samples was treated with 80% chilled acetone (1:8, *w*/*v*) by a Virtis High Speed Homogenizer for 5 min, then filtered by a vacuum and the liquids were collected. We repeated the above steps 7 times and removed the acetone by a rotary evaporator at 45 °C. We added an equal volume of methanol to the residue with mixing. Modest *n*-hexane was added to the methanol–water mixture to wash it. After centrifugation (2500× *g*, 10 min), the hexane phase was removed, and the residue was cleaned 3 times. After evaporation, the methanol was removed, and the final residue was diluted with MilliQ water saving soluble free phenolics at −20 °C.

All of the solid residues (about 4 g) from the last step were gathered, hydrolyzed with NaOH (16 mL, 4 M), and shaken for 1 h. Modest *n*-hexane was added to the mixture, after neutralizing with hydrochloric acid to pH 7.0. We removed the organic phase through centrifugation. After cleaning twice, 20% methanol/ethyl acetate was added to the water phase with the same centrifugation procedure, repeating 5 times. After evaporation, the organic regents were removed, and the final residue was diluted with MilliQ water, saving soluble free phenolics at −20 °C.

### 2.4. Total Phenolic and Flavonoid Content Measurement

The Folin–Ciocalteu colorimetric method was selected to measure the content of total phenolics in samples [29]. Briefly, both free and bound extracts were oxidized by the chemical regent mentioned in the method, and sodium carbonate neutralized the reaction. The reaction lasted 1.5 h. The absorption values were determined at 760 nm by an MRX Microplate Reader. The final data were repeated for three replications (mg GAE per 100 g) with the mean ± SD.

The total flavonoid contents of our samples were detected by the sodium borohydride/chloranil assay (SBC) by our laboratory [30], using catechin as the standard (0.1–8 mM). Briefly, the solvent of the sample was removed by a vacuum concentrator, then dissolved in tetrahydrofuran/ethanol (1:1, *v*/*v*). NaBH_4_, AlCl_3_, acetic acid solution and chloranil were added into each sample. After keeping samples at 100 °C for 1 h, vanillin and HCl were added. The absorption values were determined at 490 nm by an MRX Microplate Reader. The results were recorded as the mean ± SD for three replications (mg CE per 100 g).

### 2.5. Phytochemical Profiles Analysis by High-Performance Liquid Chromatography (HPLC)

The phytochemical profiles of soluble free and bound compounds were detected by an HPLC system [31] (Waters Corporation, Milford, MA, USA), slightly modified. A C18 column (250 × 4.6 mm, 5 μm, Sunfire) was used and the gradient mobile phase (A: Milli-Q water with 0.1% formic acid, B: acetonitrile) was indicated as below: 0–5 min (95% A), 5–12 min (95–93% A), 12–18 min (93–78% A), 18–21 min (78–55% A), 21–25 min (55–95% A), and 25–30 min (95% A) with a 280 nm assay. The column temperature was 35 °C and the velocity of flow and addition volume were 1 mL per min and 10 μL, respectively. The results were displayed as the mean ± SD for three replications (mg per 100 g).

### 2.6. Extraction of Vitamin E and Carotenoids

The vitamin E was extracted by the previous method, slightly modified [32]. In short, pistachios were ground into powder within liquid nitrogen. Then, they were added into 95% ethanol solution with sodium chloride, ascorbic acid, and pyrogallol. Next, we added KOH to saponificate the samples at 75 °C for 45 min. Adding ethyl acetate/hexane (1:9, *v*/*v*), the organic fraction was collected and then dried with N_2_. Finally, the substances were dissolved into hexane with isopropyl alcohol (1%) and methyl tert-butyl ether with butylated hydroxytoluene (1%), respectively, storing at −20 °C until analysis.

### 2.7. Vitamin E and Carotenoid Profile Analysis by HPLC

The analysis of vitamin E profile was conducted using the methods reported previously [33], with an Agilent column (250 mm × 4.6 mm, 5 μm, ZORBAX RX-SIL). The major part of the mobile phase was the *n*-hexane, followed by 0.1% acetic acid and 0.85% isopropyl alcohol (*v*/*v*). The addition volume was 20 μL. Further, 290 nm and 330 nm were selected as excitation and emission wavelengths. The vitamin E compositions of sample extracts were quantitative by relative retention time.

The carotenoid profile was determined using similar methods we have reported previously [32]. Briefly, the HPLC system consisted of a column (4.5 × 250 mm, 5 μm, YMCTM). Phase A consisted mainly of 97% methanol. The rest was ammonium acetate, butylated hydroxytoluene, methyl tert-butyl ether and butylated hydroxytoluene. Phase B and phase A were the same in composition, but in different proportions. The signal was detected under a wavelength of 450 nm. The compositions of sample extracts for carotenoids were quantified. The data on vitamin E and carotenoids were shown as μg per 100 g (mean ± SD, *n* = 3).

### 2.8. Antioxidant Activity Assay

#### 2.8.1. Determination of Oxygen-Radical-Scavenging Capacity

The determination of ORAC was assessed as reported previously [34,35]. Briefly, the extracts, Trolox standard and blank were blended with fluorescein-Na and ABAP in a 96-well plate. The intensity of fluorescence was measured using 485 nm and 535 nm by a Microplate Reader. The values of the ORAC assay were reported as micro-mol equivalent of Trolox per 100 g. The data of this assay were collected in triplicate.

#### 2.8.2. Determination of Peroxyl-Radical-Scavenging Capacity (PSC) Assay

The PSC assay was established by our research group and was conducted as reported previously [36]. In short, we added DCFH and vitamin C standard solution or sample extracts to a 96-well plate, with shaking. The fluorescence at 485 nm excitation was measured every 2 min with emission at 538 nm by Microplate Reader. The PSC value was reported as micro-mol equivalent of vitamin C per 100 g of samples. The data of this assay were collected in triplicate.

#### 2.8.3. Cellular Antioxidant Activity (CAA) Assay

The assay of CAA was developed by our laboratory [37]. CAA could measure the ability of compounds, especially phenolics and flavonoids, to prevent the oxidation of DCFH to DCF by ABAP-generated peroxyl free radicals in HepG2 cells. Briefly, HepG2 cells were placed on a 96-well microplate at the density of 6 × 10^5^/mL, with WME. After one day, cells were exposed to 100 μL WME plus DCFH-DA for 60 min at 37 °C. Then, we added 600 μM ABAP to each well with or without a PBS wash protocol. Next, the initial measured result was shown by a Microplate Reader for absorbance analysis with 538 nm and 485 nm every 5 min for 60 min. The values of this test were reported as μmol quercetin equivalents per 100 g.

### 2.9. Cytotoxicity and Antiproliferative Activity Assays

The cytotoxicity and antiproliferative activities of the samples were determined by the methylene blue assay developed in our laboratory previously [38]. Three common cancer cells were selected for this study, HepG2, Caco-2 and MDA-MB-231, respectively. First, cells in growth media (1.5 × 10^5^ cells/mL, except for MDA-MB-231 cells 1 × 10^5^/mL) were placed into a 96-well plate. After cultivation for 24 h, removing the growth medium, the DMEM media with various concentrations of pistachio extracts were added into the wells, respectively. After 24 h, the viable cells were counted to measure the cytotoxicity, which was determined by the median CC_50_ values (cytotoxicity concentration at 50% of cell death) and were recorded as milligrams per milliliter (mg/mL). The data of this assay were repeated in triplicate.

The antiproliferative activities assay was determined using the same methods we reported previously [38]. Cancer cells were (2.5 × 10^5^ cells/mL, except for MDA-MB-231 cells 2 × 10^5^ cells/ well) cultivated for 4–6 h. The various concentrations of pistachio extracts were added to a plate. Cells in each well were cultivated for 72 h. The antiproliferative activities of pistachio extracts were reported as milligrams per milliliter (mg/mL). The data of this assay were repeated in triplicate.

### 2.10. Statistical Analysis

Data among various groups were analyzed through a one-way ANOVA (IBM SPSS 24.0). If the value of p was less than 0.05, these different groups were labeled as statistically significant. CalcuSyn 2.1 (Biosoft, Cambridge, UK) was applied to analyze the dose–effect relationships. Sigmaplot software for Windows version 14.0 (Systat Software, San Jose, CA, USA.) was used to calculate the integral. All the experimental results of this paper were reported as means ± standard deviations in triplicate.

## 3. Results

### 3.1. Total Phenolics and Total Flavonoids

Data on both phenolics and flavonoids are shown in Table 1. In terms of total phenolics, there was no significant difference in the bound form of raw and roasted pistachio kernels, at 85.12 ± 3.00 and 73.11 ± 2.11 mg GAE/100 g, respectively. The contributions of the soluble free state to the total phenolics were 82% for raw pistachios and 84% for roasted, with contents of 394.8 ± 13.12 and 374.8 ± 8.47 mg GAE/100 g, respectively. This indicated the soluble free component was the main form of phenolics in both raw and roasted pistachios. At the same time, the soluble free form of raw pistachios was significantly higher than that of the roasted pistachios, which made the total phenolic content of raw pistachios also higher than that of the roasted ones (479.9 vs. 447.9 mg GAE/100g). In terms of total flavonoids, whether bound, free or total, raw pistachios had significantly higher contents than roasted pistachios.

In general, free phenolics accounted for 80% and the bound state accounted for 20% of total phenolics in both raw and roasted pistachios. For flavonoids, the free form accounted for 65–70% and the bound form accounted for 30–35% of total flavonoids in both raw and roasted pistachios. Therefore, these four phytochemical extracts, free vs. bound and raw vs. roasted, were all utilized to analyze antioxidant and antiproliferative activities next.

### 3.2. Phenolic Profiles

The phenolic profiles in pistachios, analyzed by the HPLC method, are shown in Table 2. There were three phenolic acids, two flavonoids and one other polyphenol in raw and roasted pistachios. Gentisic acid was the main substances in raw pistachios, with a content of 120.76 ± 70.81 mg/100 g (69% of total detected), followed by catechin (21.10 ± 1.49 mg per 100 g, 12% of total detected). In contrast to raw pistachios, there was more gentisic acid and catechin in roasted pistachios, with increases of 1.76-fold and 1.60-fold, respectively. In general, roasted pistachios had significantly higher phenolics than the raw pistachios, except for protocatechuic acid (similar) and epigallocatechin gallate (lower).

### 3.3. Profiles of Vitamin E and Carotenoids

The profiles of vitamin E and carotenoids in pistachio kernels detected through the HPLC method are shown in Table 3. In general, four isomers of vitamin E and three carotenoids were detected in raw and roasted pistachio kernels. The roasted pistachios had significantly higher contents of α-tocotrienol and β-tocopherol when compared with raw pistachios. The contents of γ-tocopherol and γ-tocotrienol were similar in both raw and roasted pistachios. In addition, β- and γ-tocopherols were the major isomers of vitamin E in both raw and roasted pistachios, accounting for 87.89% (raw) and 87.30% (roasted) of the total vitamin E.

As for carotenoids, there were lutein, zeaxanthin and β-carotene in pistachios, detected by the HPLC assay. Raw pistachios had significantly higher contents of lutein and β-carotene when compared with roasted pistachio kernels, except for zeaxanthin. Lutein was the major carotenoid in pistachios, accounting for 90.9% (raw) and 89.3% (roasted) of the total. In contrast to roasted pistachios, raw pistachios had significantly higher lutein content, by 1.44-fold (1366.7 ± 76 μg/100 g).

### 3.4. Total Antioxidant Activity

#### 3.4.1. ORAC and PSC Assays

**Figure 1:**
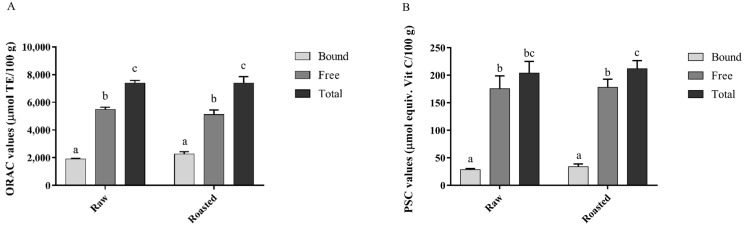
Total antioxidant activity of pistachios (raw and roasted) tested by ORAC (**A**) and PSC (**B**) assays. Values with different letters in each bar are contrasting (*p* < 0.05) significantly.

The ORAC and PSC measurements were applied for analyzing the antioxidant activity of pistachio kernels. The data are reported Figure 1. According to ORAC values in bound, free and total forms between raw and roasted pistachios, there was no significant difference. The ORAC values of the free form in raw (5484.56 ± 161 check the number μmol TE/100 g) and roasted pistachios (5111.46 ± 325 μmol TE/100 g) were significantly higher than the bound form, accounting for 75% and 70% of total, respectively. The trends were similar in the PSC assay in comparison to the ORAC assay, indicating the consistency of both methods. The PSC values of the free form in raw and roasted pistachios were significantly higher than the bound form, accounting for 86% and 80% of the total, respectively. Overall, there was consistency between the antioxidant activities determined by both ORAC and PSC assays.

#### 3.4.2. CAA Assay

**Figure 2:**
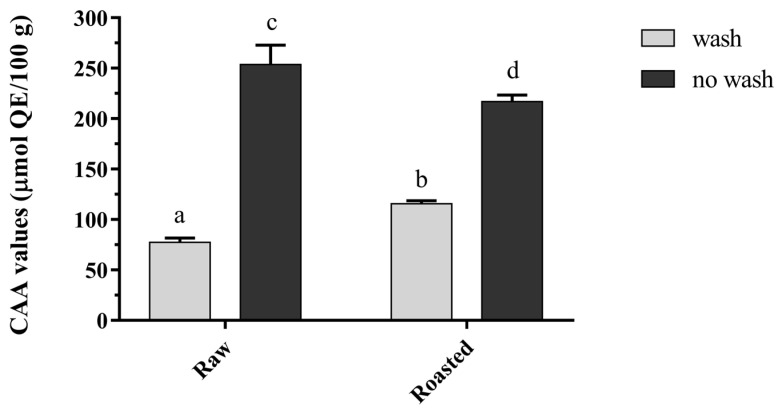
Cellular antioxidant activity (CAA) of pistachios (raw and roasted, free form). Values with different letters in each bar are contrasting (*p* < 0.05) significantly.

The CAA assay was applied to evaluate the antioxidant capacity at the cellular level. The determinations were divided into two parts, PBS wash or no PBS wash, as shown in Figure 2. and Table 4. Both of the free forms of the two pistachio extracts were taken into account, since the pre-experiment showed the bound form extracts held little cellular antioxidant activity. The contents of bioactivities in bound-form extracts were significantly lower than free-form ones (see data above for total phenolics, ORAC values and PSC values). According to CAA results, both raw and roasted pistachio extracts exhibited cellular antioxidant activity, inhibiting peroxyradical radical-induced oxidation, with CAA values of 77.39 ± 4.25 (wash) and 253.71 ± 19.18 (no wash) μmol QE/100 g for raw pistachios and 115.62 ± 3.02 (wash) and 216.76 ± 6.6 (no wash) μmol QE/100 g for roasted pistachios. In terms of different procedures, CAA values without PBS wash were significantly higher than with PBS wash, by 3.28-fold (raw) and 1.87-fold (roasted) increases, respectively. The variances in CAA values using the protocols with or without PBS wash were related to the level of cellular absorption and membrane association of sample extracts, because the compounds which were loosely linked to the cell membrane would be removed with PBS wash [37]. In terms of results, both raw and roasted pistachio extracts were absorbed loosely to the cell membrane to some extent, with cell uptakes of 30.5% and 53.34%, respectively.

### 3.5. Antiproliferative Activity in Cancer Cells

**Figure 3:**
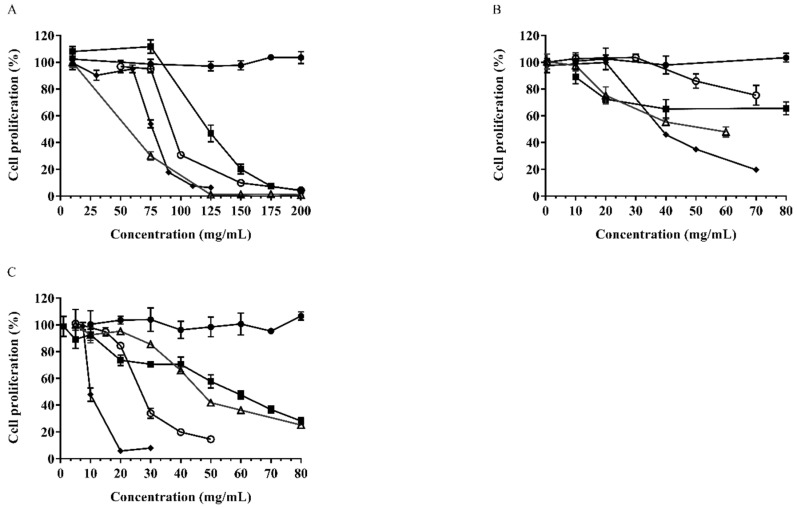
Percent inhibition of cancer cells’ proliferation by pistachios (raw and roasted). (**A**): HepG2 cells, (**B**): Caco-2 cells, (**C**): MDA-MB-231 cells. (●) control; (△) raw–bound; (○) raw–free; (■) roasted–bound; (◆) roasted–free.

The results of antiproliferative activity in cancer cells are shown in Figure 3 and Table 5. The lower median effective dose (EC_50_) value represented higher antiproliferative ability and the higher median cytotoxic dose (CC_50_) value represented lower cytotoxicity. The ratio of CC_50_ to EC_50_ was reported as the selectivity index (SI) value. A higher SI value indicates a higher specific antiproliferative activity [31]. Raw and roasted pistachios’ phytochemical extracts exhibited potent antiproliferative activities. The free-form extracts of roasted pistachios exhibited relatively high antiproliferative capacity towards HepG2, Caco-2 and MDA-MB-231 cells in dose-dependent manners. In terms of raw pistachios, the bound-form pistachio extracts had higher antiproliferative activities against HepG2 and Caco-2 cancer cell growths, with EC_50_ values of 47.84 ± 5.44 and 51.49 ± 4.4 mg/mL, respectively, when compared with the free form. However, there was no detected antiproliferative effect of raw pistachio extracts on MDA-MB-231 cell lines (SI < 2). As for roasted pistachios, the EC_50_ of free extracts of roasted pistachios were 34.73 ± 1.64 mg/mL (HepG2), 36.66 ± 3.3 (Caco-2) and 7.41 ± 0.82 (MDA-MB-231) mg/mL, with high SI values of 4.2, 5.43 and 8.01, respectively, which indicated the superior potential for antiproliferative activities against these cancer cells. At the same time, the bound-form extracts of roasted pistachios also exhibited potent antiproliferative activities towards HepG2, Caco-2 and MDA-MB-231 cancer cells, with SI values of 3.17, 3.81 and 2.42, respectively. Overall, in terms of antiproliferative activities against the three cancer cell lines, roasted free extracts had much higher anticancer activities among all extracts tested. It is worth pointing out that free extracts of roasted pistachios exhibited exceptionally high activity against human breast cancer MDA-MB-231 cells, with an EC_50_ of 7.41 ± 0.82 mg/mL and SI of 8.01.

## 4. Discussion

Previous studies had reported that pistachios were rich in both non-polar and polar bioactive compounds [12,39,40]. These abundant and various phytochemicals could exert positive health effects. In this study, we explored the phytochemical profiles, antioxidant activity, cellular antioxidant activity, and antiproliferative activities against human liver cancer HepG2, human colon cancer Caco-2, and human breast cancer MDA-MB-231 cells of raw and roasted pistachios for the first time.

Vitamin E and carotenoid profiles of pistachios indicated that pistachios were rich in lipids with antioxidant properties, especially β-tocopherol, γ-tocopherol and lutein. Previous studies have determined that γ-tocopherol is the primary component of vitamin E in California pistachios [12,40]. However, our study showed that pistachios were not only rich in γ-tocopherol but also in β-tocopherol. Grace et al. [11] found the α-tocopherol concentration of Kerman pistachios was 5.34 mg per 100 g. Additionally, the content of γ-tocopherol was 39.71 mg per 100 g. The δ- tocopherol content was the lowest, at only 1.68 mg per 100 g. In comparison, the contents of vitamin E that we obtained were significantly lower than in previous studies. The inconsistency existing among these studies may stem from different analytical methods and place of origin. As for carotenoids, we reported pistachios contain lutein, zeaxanthin, and β-tocopherol, and lutein is the major carotenoid in pistachios. These results were consistent with research published previously [12,40]. Simona [12] et al. reported that there were significant xanthophyll carotenoids in pistachios (only the nut), which were lutein and zeaxanthin.

According to antioxidant activity and the contents of antioxidant components, phenolics and flavonoids of the phytochemical extracts were significantly higher in the raw pistachios than in the roasted pistachios, except for the bound form of raw and roasted pistachios, which were similar. The levels of antioxidant activities (ORAC, PSC and CAA) of the phytochemical extracts correlated well with the contents of active components. Although the total phenolics and flavonoids of raw pistachios were higher than those of roasted pistachios, we found there was more gentisic acid and catechin in roasted pistachios than in the raw pistachios. It has been shown that the roasting procedure could enhance total phenolics and the contents of some phenolics such as protocatechuic acid and catechin in roasted pistachios (single-roasted or double-roasted), which were significantly higher than in raw pistachios [41]. This may be due to heat processing promoting the release of phenolics [42,43]. At the same time, the differences between components may not be shown by regular chemical assays, which cannot mimic the complexity of biological systems; however, these activities could be detected by more physiologically relevant assays such as the CAA assay and antiproliferative activity assay.

The CAA assay showed superior biological relevance, because CAA does not only address chemical antioxidant reactions by quenching free radicals at the cellular levels, but also considers cellular uptake, cellular physiological conditions, and metabolism conditions [37]. It can be seen that the CAA assay was a more effective and accurate method for determining cellular antioxidant activity than the traditional chemical antioxidant activity assays. However, there were no reports in the literature to evaluate the cellular antioxidant activity of pistachios using the CAA assay. We reported here for the first time that free-form extracts of pistachios exhibited cellular antioxidant activity. CAA values in roasted pistachios with PBS wash were significantly higher than in the raw pistachios, indicating that cellular uptake may be increased in roasted pistachios, which may be related to the changes in substances. This phenomenon is worth further research.

Antiproliferative activity assays have been commonly used to screen anticancer activities of anticancer drugs, natural products, food components, and dietary supplements [39,44,45,46,47,48]. Here, we reported the antiproliferative effects of pistachio extracts against three HepG2 (human liver cancer), Caco-2 (colon), and MDA-MB-231 (breast) cells. The free extract of roasted pistachios had potent antiproliferative activities against all three cancer cell lines at the concentration tested, without cytotoxicity. However, previous research reported that the extracts of pistachios (variety Kerman) held almost no antiproliferative activities against HepG2 and Caco-2 cells [39]. However, in 2018, Reboredo-Rodríguez [49] et. al reported that pistachio (variety Sirora) kernel extracts caused a decrease in cell viability in MCF-7 breast cancer cells. It was also reported that the fermented extracts of pistachios exhibited antiproliferative effects in human colon adenocarcinoma LT9728 and HT2945 cells [50]. Besides the kernels of pistachios, the hull phytochemical extracts of pistachios [26,51] were reported to have apoptosis-induction and angiogenesis potential in six human cancer cells, namely HT-29 (colon cancer), HCT-116 (colon cancer), MCF-7 (breast adenocarcinoma), H23 (lung adenocarcinoma), HepG2 (liver cancer), Ca Ski (cervical cancer), and one normal fibroblast (BJ-5ta). Among the six cell lines, the MCF-7 cells were the most sensitive cells and the HepG2 cells were the most resistant. Our work is consistent with published studies. Further research on the anticancer mechanism of action of pistachios is needed.

## 5. Conclusions

Our results provide new knowledge about the phytochemical profiles (total phenolics, flavonoids, vitamin E and carotenoids), antioxidant activity, cellular antioxidant activity and antiproliferative activity of raw and roasted pistachios. It is shown that the roasting of pistachios could produce a series of beneficial phytochemical changes, leading to enhanced biological activity. Pistachios are a nutrient-dense food containing a unique profile of good-quality protein, fats, minerals, vitamins, and antioxidants, such as carotenoids and polyphenols, with cellular antioxidant activity. Dietary Guidelines for Americans 2020–2025 suggested including nuts as a health dietary pattern. Further research on antiproliferative activity and mechanisms of action of free-form extracts of roasted pistachios, and more biological activities related cellular antioxidant activity and oxidative stress, are worthy of further investigation.

## Figures and Tables

**Table 1:** Total phenolics contents of pistachios (raw and roasted).

Pistachios	Total Phenolics (mg GAE/100 g)			Total Flavonoids (mg CE/100 g)		
	Bound (Percentage of Total)	Free (Percentage of Total)	Total	Bound (Percentage of Total)	Free (Percentage of Total)	Total
Raw	85.12 ± 3.00 ^a^ (18)	394.8 ± 13.12 ^c^ (82)	479.9 ± 10.2 ^e^	62.56 ± 2.78 ^a^ (35)	115.8 ± 13.3 ^c^ (65)	178.4 ± 10.6 ^e^
Roasted	73.11 ± 2.11 ^a^ (16)	374.8 ± 8.47 ^b^ (84)	447.9 ± 9.4 ^d^	42.76 ± 2.73 ^b^ (30)	101.4 ± 4.64 ^d^ (70)	144.1 ± 7.4 ^f^

Values with different letters in common are contrasting (*p* < 0.05) significantly.

**Table 2:** Phenolic profiles of pistachios (raw and roasted).

Compounds	Raw (mg/100 g)			Roasted (mg/100 g)		
	Bound	Free	Total	Bound	Free	Total
Phenolic acids						
Gallic acid	3.45 ± 0.30 ^a^	9.88 ± 0.11 ^b^	13.33 ± 0.22 ^c^	2.05 ± 0.09 ^d^	16.05 ± 1.04 ^e^	18.11 ± 1.07 ^f^
Protocatechuic acid	0.23 ± 0.01 ^a^	2.13 ± 0.14 ^bc^	2.36 ± 0.14 ^c^	0.89 ± 0.04 ^ab^	2.37 ± 1.22 ^c^	3.25 ± 1.25 ^c^
Gentisic acid	3.09 ± 0.30 ^a^	117.67 ± 70.74 ^b^	120.76 ± 70.81 ^b^	9.25 ± 0.66 ^a^	202.90 ± 11.88 ^c^	212.15 ± 12.34 ^c^
Flavonoids						
Catechin	0.17 ± 0.01 ^a^	20.93± 1.48 ^a^	21.10 ± 1.49 ^d^	0.35 ± 0.03 ^b^	33.44 ± 3.00 ^c^	33.80 ± 3.03 ^c^
Epigallocatechin gallate	3.48 ± 0.19 ^a^	14.31 ± 1.49 ^b^	17.78 ± 1.66 ^c^	N/A	6.26 ± 0.79 ^d^	6.26 ± 0.79 ^d^
Other polyphenols						
Catechol	0.27 ± 0.02 ^a^	N/A	0.27 ± 0.02 ^a^	0.57 ± 0.09 ^b^	7.30 ± 0.34 ^c^	7.88 ± 0.31 ^d^

Values with different letters in common are contrasting (*p* < 0.05) significantly.

**Table 3:** Vitamin E and carotenoids profiles of pistachios (raw and roasted) (independent T-test).

Compounds	Vit. E (μg per 100 g)		Compounds	Carotenoids (μg per 100 g)	
	Raw	Roasted		Raw	Roasted
α-Tocotrienol	52.58 ± 4.20 ^a^	85.64 ± 4.74 ^b^	lutein	1366.7 ± 76 ^a^	951.2 ± 51 ^b^
β-Tocopherol	1252.7 ± 16 ^a^	1346.7 ± 7.9 ^b^	zeaxanthin	42.05± 9.46 ^a^	43.49 ± 3.24 ^a^
γ-Tocopherol	918.3 ± 85.3 ^a^	1028.6 ± 2.3 ^a^	β-carotene	94.96 ± 6.22 ^a^	70.39 ± 1.33 ^b^
γ-Tocotrienol	246.6 ± 15.2 ^a^	260.0 ± 1.8 ^a^	Total carotenoids	1503.64 ± 69 ^a^	1065.04 ± 55 ^b^
Total Vit. E	2470.2 ± 89 ^a^	2720.9 ± 1.0 ^b^			

Values with different letters in common are contrasting (*p* < 0.05) significantly.

**Table 4:** Vitamin E and carotenoid profiles of pistachios (raw and roasted) (independent T-test).

Free	CAA Values (μmol QE per 100 g)	
	Wash	No Wash
Raw	77.39 ± 4.25 ^a^	253.71 ± 19.18 ^b^
Roasted	115.62 ± 3.02 ^c^	216.76 ± 6.60 ^d^

Values with different letters in common are contrasting (*p* < 0.05) significantly.

**Table 5:** Vitamin E and carotenoids profiles of pistachios (raw and roasted) (Independent T-test).

Pistachios		Raw (mg/mL)				Roasted (mg/mL)			
Cells		Bound	SI	Free	SI	Bound	SI	Free	SI
HepG2	EC_50_	47.84 ± 5.44 ^b^	2.41	100.72 ± 4.72 ^c^	2.01	117.48 ± 6.11 ^d^	3.17	34.73 ± 1.64 ^a^	4.20
	CC_50_	115.49 ± 2.20 ^a^		202.73 ± 6.46 ^c^		372.60 ± 0.76 ^d^		145.96 ± 7.14 ^b^	
Caco-2	EC_50_	51.49 ± 4.4 ^a^	2.20	117.13 ± 2.9 ^b^	2.24	151.67 ± 14.8 ^c^	3.81	36.66 ± 3.3 ^a^	5.43
	CC_50_	113.44 ± 8.0 ^a^		262.87 ± 24.0 ^b^		578.53 ± 44.5 ^d^		198.92 ± 20.4 ^c^	
MDA-MB-231	EC_50_	51.04 ± 0.69 ^c^	1.85	28.12 ± 1.43 ^b^	1.50	57.61 ± 2.18 ^d^	2.42	7.41 ± 0.82 ^a^	8.01
	CC_50_	94.20 ± 3.91 ^c^		42.15 ± 6.71 ^a^		139.22 ± 1.31 ^d^		59.39 ± 2.96 ^b^	

SI, selectivity index = CC_50_/EC_50_. Values with different letters in common in each raw are contrasting (*p* < 0.05) significantly.

## Data Availability

Not applicable.
